# Acute Cytomegalovirus (CMV) Infection Associated with Hemophagocytic Lymphohistiocytosis (HLH) in an Immunocompetent Host Meeting All Eight HLH 2004 Diagnostic Criteria

**DOI:** 10.7759/cureus.1070

**Published:** 2017-03-02

**Authors:** Alex K Bonnecaze, Wesley G Willeford, Peter Lichstein, Jill Ohar

**Affiliations:** 1 Department of Internal Medicine, Wake Forest University Baptist Medical Center; 2 Department of Pulmonary, Critical Care, Allergy and Immunologic Diseases, Wake Forest University Baptist Medical Center

**Keywords:** hemophagocytic lymphohistiocytosis, hlh, cytomegalovirus, cmv, immunocompetent, hlh-2004

## Abstract

Hemophagocytic lymphohistiocytosis (HLH) is a rare and often deadly syndrome characterized by severe inflammation and cytokine dysregulation. The disease is defined by the HLH-2004 criteria, requiring five of eight findings, and is further differentiated into either primary or secondary causes. Primary HLH tends to be of genetic etiology, while secondary HLH results from other insults such as infection. Secondary HLH is most commonly associated with viral infections in immunocompromised patients. Acute cytomegalovirus (CMV) associated HLH in the immunocompetent host is exceedingly rare and only documented in four case reports to date. We describe the fifth documented case of CMV-associated HLH in an immunocompetent patient, and furthermore, we demonstrate that this patient is the first published case of its type to satisfy all eight of HLH-2004 criteria.

## Introduction

Hemophagocytic lymphohistiocytosis (HLH) is a devastating and rare disorder with an incidence of only 1.2 cases per million patients per year, with a startlingly high mortality rate of 47% [1]. This disease entity is characterized by defective natural killer cell cytotoxicity, which results in an inappropriately robust activation of macrophages and leads to engulfment of other blood cells. The resulting syndrome is one of high fever, hepatosplenomegaly, lymphadenopathy, and cytopenias. Tseng, et al. found that 60% of 96 patients who met the 2004 HLH diagnostic criteria had leukopenia, 20% had hepatosplenomegaly, and 20% developed jaundice [2]. Due to severe fevers, HLH is frequently mistaken for a septic syndrome, leading to profound delays in time to diagnosis of this rare condition. Tseng, et al. also found that the median time to diagnosis of HLH was 34.5 days [2]. Other clinical presentations reported in the literature include fever of unknown origin, hepatitis and acute liver failure, and neurological abnormalities [3].

HLH is traditionally divided into primary and secondary causes. Cases of primary HLH result from genetic abnormalities that lead to defective protein function in the cytolytic secretory pathway, and 80% of these cases present in the first year of life [3]. Examples of genetic conditions associated with primary HLH include Griscelli syndrome 2 (GS2) and Chediak-Higashi disease (CHD). Secondary HLH is typically caused by an exogenous insult in an immunocompromised patient. Common causes are Epstein-Barr virus (EBV), cytomegalovirus (CMV), human immunodeficiency virus (HIV), hepatitis A virus, Herpesviridae, and bacterial, parasitic, and fungal etiologies [3]. CMV-associated HLH has been reported in a variety of immunocompromised states including solid organ transplantation, infection, and autoimmune disease [1]. CMV-associated HLH in an immunocompetent patient has only been reported in the literature on four other occasions [1-2, 4-5]. HLH is defined by meeting at least five of eight HLH 2004 diagnostic criteria [6]. These criteria include: the presence of fever, splenomegaly, cytopenias affecting greater than or equal to two of three lineages in the peripheral blood, hypertriglyceridemia (≥ 265 mg/dL), hemophagocytosis in the bone marrow, spleen, or lymph nodes, soluble CD25 > 2400 U/mL, low or absent natural killer cell (NK-cell) activity, and ferritin ≥ 500 μg/L. Since the introduction of these guidelines, mortality due to HLH has dropped by 30-35%, and this is likely due to early recognition [1].

## Case presentation

**Figure 1:**
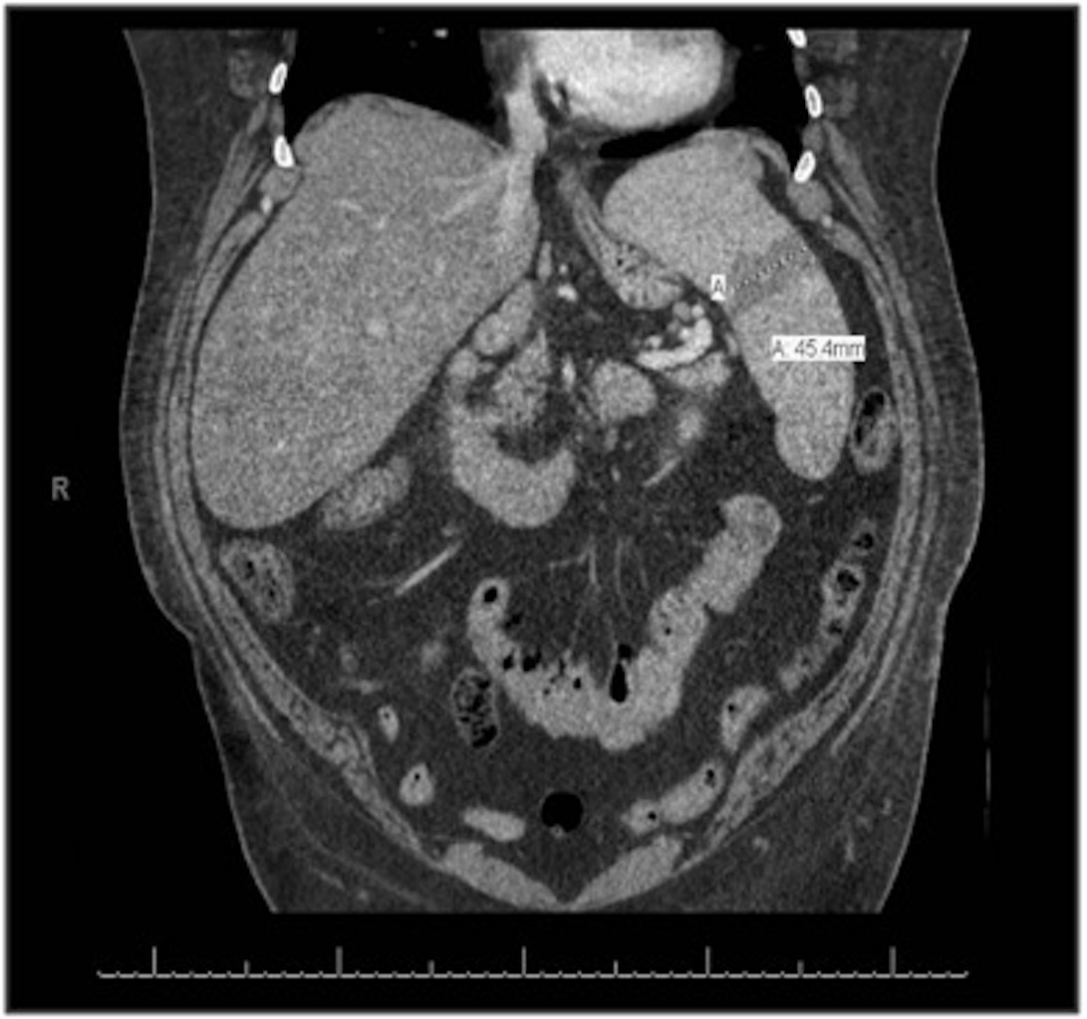
Acute splenic infarction on admission - CT-abdomen imaging. Hepatosplenomegaly and abdominal lymphadenopathy were also noted.

A 39-year-old morbidly obese female with poorly-controlled type II diabetes mellitus, hypertension, and obstructive sleep apnea presented with a four-day history of night sweats, fevers, and progressive abdominal pain without nausea, vomiting, or diarrhea. Initial physical examination revealed a temperature of 38.9°C, heart rate of 113 beats per minute, acanthosis nigricans of the posterior neck, and diffuse epigastric tenderness. Initial laboratory values included a creatinine of 0.86 mg/dL, lactic acid of 2.4 mMol/L, lipase of 48 u/L, LDH of 664 u/L, ferritin of 1454 ng/mL, and normal transaminases. A computed tomography (CT) scan of the abdomen and pelvis with contrast was obtained and revealed an acute wedge-shaped splenic infarction, hepatosplenomegaly, abdominal lymphadenopathy, and groundglass opacities in the right lower lung lobe concerning for pneumonia (Figure 1). Additional tests shortly after admission included a negative HIV antibody test, a negative antinuclear antibody screen, and hepatitis screen revealing positive hepatitis-B core antibody (Table 1).

Intravenous moxifloxacin was given to treat a presumptive pneumonia, and antibiotics were later escalated to piperacillin-tazobactam after failure to improve. Vancomycin was added several days later out of concern for possible infectious endocarditis on transthoracic echocardiogram (TTE). The patient denied intravenous drug usage; however, she did acknowledge a history of recurrent dental infections. Subsequent transesophageal echocardiogram (TEE) was delayed, but ultimately, it did not demonstrate any valvular lesions.

On the fifth day of admission, the patient suffered acute hypoxemic respiratory failure believed to be secondary to pneumonia and developing acute respiratory distress syndrome. She was transferred to the medical intensive care unit for more aggressive care and diuresis. Despite these interventions, the patient developed worsening respiratory failure and shock refractory to crystalloid fluid administration. She required intubation and inotropic support. The patient continued to be febrile without improvement of respiratory failure despite negative blood cultures and greater than 10 days of broad-spectrum antibiotics. Piperacillin-tazobactam was changed to meropenem, given worsening clinical picture and concern for multi-drug-resistant organisms, given fever of unknown origin and her history of dental infections.

Factors that suggested HLH at this point included an acute elevation of ferritin, persistent fever, splenomegaly, and new-onset hypertriglyceridema (617 mg/dL). By the 20th day of hospitalization, multiple viral studies had been obtained, all of which were unremarkable other than a positive CMV immunoglobulin M (IgM) and CMV immunoglobulin G (IgG). A thorough workup (Table 1) was not suggestive of other etiologies. Given the finding of a positive CMV IgM, a CMV-DNA polymerase chain reaction (PCR) was obtained which showed a viral load of 119,611 c/mL. A bronchial alveolar lavage (BAL) was performed due to persistent respiratory failure and pneumonia, and this yielded a positive CMV PCR. A diagnosis of acute CMV infection with highly probable CMV-pneumonia was made. While histopathologic diagnosis of CMV-pneumonia requires visualization of viral inclusion bodies in lung or transbronchial tissue, it is generally accepted that a clinical diagnosis may be made when viral pneumonia is suspected in addition to a positive BAL CMV PCR [7].

**Figure 2:**
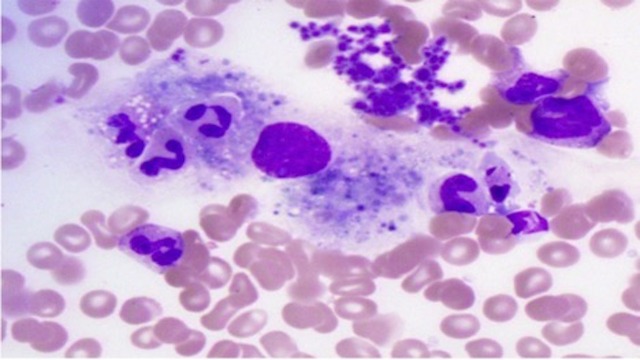
Hemophagocytosis on bone marrow biopsy.

Given persistent fevers despite therapy and a markedly elevated ferritin, which continued to increase (10,000 ng/mL), hematology-oncology was consulted. A bone marrow biopsy was performed and revealed scattered hemophagocytosis (Figure 2). A natural killer (NK) cell function assay revealed absent activity. Soluble CD25 receptor was elevated at 3611 U/mL. At this time, the patient met all eight HLH diagnostic criteria. Additionally, she had demonstrated acute CMV infection via robust viremia and positive CMV PCR on BAL.

Intravenous (IV) ganciclovir therapy was initiated and dosed for renal impairment once CMV viral load was found to be markedly elevated in addition to a positive BAL CMV PCR. Clinically, the patient failed to improve with IV ganciclovir; however, the CMV viral load significantly decreased shortly after antiviral therapy was started (119,611 IU/ml prior to therapy, 2402 IU/ml five days after therapy, and < 137 IU/ml 11 days after therapy).

Upon diagnosis of HLH, the patient was started on IV dexamethasone therapy (four week taper) without concomitant cytotoxic therapy. While chemotherapy, such as etoposide, is often used to treat HLH, the consulted hematology team felt the patient was a poor candidate for this. Clinical improvement was noted within several days of initiating IV dexamethasone. The patient eventually recovered after suffering several complications during therapy, including an episode of Proteus mirabilis septicemia secondary to central venous catheter line infection, multiple intubations, and acute renal failure secondary to acute tubular necrosis, which required renal replacement therapy. She was discharged on hospital day 58.

Her outpatient course was complicated by poor therapeutic adherence, post-traumatic stress disorder, and hypoventilation secondary to obesity and obstructive airway disease. She went on to require placement of a tracheostomy due to hypoventilation. She continues to follow-up with oncology and her primary care provider. Informed consent was obtained from the patient for this study.

 

**Table 1:** Summary of inpatient laboratory and imaging findings. Hepatitis B virus - HBV, Anti-neutrophil cytoplasmic antibodies - ANCA, Complete blood count - CBC.

Lab	Value	Lab	Value
Respiratory Virus Panel (RVP)	Negative	SSA/SSB Antibodies	Negative
Hepatitis Panel	Negative other than HBV Core Ab	Transesophageal Echocardiogram	No valvulopathy
HIV	Negative	Antiphospholipid Antibody	Negative
Rheumatoid Factor (RF)	Negative	Rocky Mountain Spotted Fever & Lyme Serology	Negative
ANCA	Negative	Ferritin	10,253 ng/mL
CMV IgM	Positive	Triglycerides	402 mg/dL
CMV IgG	Positive	Tmax	103.9° F
CMV PCR	119,611 c/mL	CBC	Hemoglobin 8.5 g/dL Platelets 78,000
Bronchial Alveolar Lavage (BAL) CMV PCR	Positive	BAL HSV PCR	Negative
BAL Aspergillus/Pneumocyst/Legionella	Negative	Bone Marrow Biopsy	Hemophagocytosis
Natural Killer Cell (NK) Activity	Absent	Splenomegaly	Present
Acid Fast/Fungal Blood Cultures	Negative	Soluble CD25	3611 U/mL

## Discussion

Hemophagocytic lymphohistiocytosis, defined by the HLH-2004 criteria, represents a syndrome of cytokine dysregulation and severe inflammatory response. Additionally, it is one of only several diseases featuring a markedly elevated ferritin level in addition to adult onset Still’s disease and lymphoma. While HLH primarily affects immunocompromised patients, it is also possible for immunocompetent individuals to be affected. Even more rare is CMV-associated HLH in the immunocompetent patient, which has been documented only four times in the literature. Tsuda, Hot, and Tseng all report cases featuring positive CMV serology and bone marrow biopsy revealing hemophagocytosis; however, they did not report measuring NK-cell activity or CD 25 levels [2, 4-5]. Atim-Oluk, et al. argue their case is the first of its kind to diagnose HLH without bone marrow biopsy; however, they also did not measure NK-cell activity and CD 25 levels [1]. This case also had potential overlap with syndromes such as adult onset Still’s disease given the lack of aforementioned studies and a bone marrow biopsy. None of these cases reported finding CMV in multiple body fluids, compared to our patient who demonstrated CMV in both blood and alveolar lavage fluid.

The patient presented above met all eight of the HLH 2004 diagnostic criteria with an elevated ferritin to 10,253 ng/mL, triglycerides of 402 mg/dL, anemia and thrombocytopenia, high fevers, absence of natural killer cell activity, soluble CD25 of 3611 U/mL, confirmed splenomegaly by computed tomography scan (Figure 1), and evidence of hemophagocytosis seen on bone marrow biopsy (Figure 2). Furthermore, CMV as the etiology for our case is better supported than the previously mentioned cases (Table 2). Tsuda, et al. had only positive values for CMV IgG and IgM; Hot, et al. had positive CMV IgM and CMV PCR revealing 41,000 viral copies/mL; Tseng, et al. did not specify in their paper; and Atim-Oltuk, et al. had positive values for CMV IgG, IgM, and a CMV PCR with 19,000 viral copies/mL. Our patient demonstrated positive values for CMV IgG, IgM, CMV PCR (119,611 copies/mL), and a bronchial alveolar lavage with qualitative CMV PCR positivity.

Despite severe CMV infections traditionally affecting neonatal and immunocompromised patients, disease in immunocompetent patients is likely underestimated. While worldwide CMV seropositivity ranges from 40-100%, the vast majority of healthy adults exposed to the virus experience an asymptomatic course or mild mononucleosis-like symptoms [7]. Acute CMV viremia in immunocompetent individuals has been documented to cause colitis, meningitis, encephalitis, myelitis, and bone marrow suppression [8]. One possible theory as to why these individuals are affected is that common conditions, such as poorly controlled diabetes mellitus and kidney disease, may create some degree of immune dysfunction. While immunocompromised individuals are traditionally thought as those suffering specific genetic disorders, taking immune-suppressive drug therapy, or suffering systemic infections such as HIV, many chronic conditions likely create a degree of immunosuppression that is not accounted for. Our patient suffered from poorly controlled diabetes mellitus in addition to morbid obesity, thus potentially creating some degree of functional immune deficiency. This theory would also explain why nearly 400 reports of seemingly healthy individuals suffering severe CMV complications exist [9]. While relative immunodeficiency is difficult to assess, it should be noted our patient did have a normal level of IgA, IgM, and IgG. Future research examining correlations between specific comorbidities and severity of CMV infection in immunocompetent patients would provide further insight to this issue. While it is highly probable that our patients’ HLH was induced from her acute multi-organ CMV infection, neither our case nor the prior documented cases can definitively prove this.

One of the more interesting findings in our case was the presence of splenic infarctions. Justo, et al. performed a meta-analysis that revealed that thrombosis in the setting of uncontrolled CMV infection has been mentioned in the literature almost 100 times [10]. The article goes on to state that deep venous thrombosis, pulmonary embolism, and splanchnic vein thrombosis are the most common thromboses associated with acute CMV infection, but splanchnic venous thrombosis is more common in immunocompetent patients.

With regard to our patient’s therapeutic regimen, she was given intravenous gangciclovir when high levels of viremia were discovered. While there is no evidence for IV gangiclovir in acute primary CMV infection, benefit does exist in cases of CMV pneumonia, which our patient likely had, given the BAL findings [7]. Once the final diagnosis for HLH was determined, she was given high dose dexamethasone (without etoposide). Per guidelines from the Histiocyte Society (HLH-94), therapy traditionally involves “remission treatment” consisting of eight weeks etoposide and dexamethasone. Further therapy varies based on subtype of HLH and whether or not remission occurs [1].

**Table 2:** Prior cases of CMV-associated HLH infection in immunocompetent hosts.

Author	Age/Sex	Diagnosis of CMV	Diagnostic Criteria for HLH
Tsuda, Shirono, et al., 1996 [5]	21M	(+) CMV IgG and IgM	Fever, Splenomegaly, Cytopenia, Ferritin 1314, Positive BMB
Hot, et al., 2008 [4]	32F	(+) CMV IgM, CMV PCR 41,000	Fever, Splenomegaly, Cytopenia, Ferritin 88,300 ng/mL, Positive BMB
Yu-Tzu Tseng, et al., 2011 [2]	N/A	Generic Criteria	Reportedly met HLH2004 criteria and BMB+ (No further details)
Atim-Oltuk, et al., 2013 [1]	48F	CMV IgG/IgM (+), CMV PCR 19,000	Fever, Splenomegaly, Ferritin 40,000 ng/mL, Cytopenia, Hypertriglyceridemia

## Conclusions

CMV-induced HLH in the immunocompromised patient is a rare disease, but in an immunocompetent patient, the diagnosis is rarer still. To date, our case represents only the fifth report of this particular scenario. Furthermore, our case is the only one to meet all eight of the HLH 2004 diagnostic criteria, and it is the only one to demonstrate CMV by PCR and immunologically in the blood and by PCR in an affected organ system. Clinicians should additionally consider other comorbidities, such as poorly controlled diabetes, which may lead to a lesser degree of immunodeficiency in critically ill patients. Other cases may be yet unpublished, but due to the rarity, rapidity, and severity of HLH, this particular syndrome may be underrepresented in the literature.
